# Comparison of the Effect of Ketamine and Dexmedetomidine Combined with Total Intravenous Anesthesia in Laparoscopic Cholecystectomy Procedures: A Prospective Randomized Controlled Study

**DOI:** 10.1155/2022/1878705

**Published:** 2022-07-21

**Authors:** E. Efe Mercanoglu, N. Girgin Kelebek, G. Turker, H. Aksu, M. Ozgur, Z. Karakuzu, S. Turkcan, B. Ozcan

**Affiliations:** Uludag University, School of Medicine, Bursa, Turkey

## Abstract

This randomized prospective clinical study aimed to investigate the effects of dexmedetomidine or ketamine administration to total intravenous anesthesia (TIVA) on postoperative analgesia in subjects undergoing elective laparoscopic cholecystectomy procedures. 90 adults, American Society of Anesthesiologists (ASA) physical status 1 and II patients, who underwent elective laparoscopic cholecystectomy procedures were included in the study and randomized into three groups equally. Remifentanil, propofol, and rocuronium infusions were used for TIVA guided by the bispectral index. In group KETA, 10 *μ*g/kg/min ketamine was added to TIVA before surgery, and in group DEX, 0.5 *μ*g/kg/h dexmedetomidine was added to TIVA before surgery. Normal saline infusions were infused in the control group. Postoperative analgesia was provided with intravenous patient-controlled analgesia (PCA) morphine (1 mg bolus morphine, 5 min lockout time). Hemodynamic parameters, scores of visual analogue scale (VAS) for pain, rescue morphine requirements, and side effects such as sedation, nausea, and vomiting were recorded for 48 hours after surgery. Postoperative first analgesic requirement time was longer in group KETA (*P* < 0.001), and it was longer in group DEX than in the control group (*P* < 0.001). Pain scores were lower in group KETA and group DEX than in the control group at all corresponding times throughout the 48 h period of observation. Intravenous PCA morphine consumptions were higher in the control group than in group KETA (*P* < 0.001 for all followed-up times), and they were higher in group DEX than in group KETA (*P* < 0.001 for all followed-up times). It is concluded that the use of dexmedetomidine or ketamine infusions can be suitable as an additive for TIVA in the intraoperative period. Furthermore, the addition of both drugs to the TIVA protocol may improve postoperative pain relief and decrease opioid consumption.

## 1. Introduction

In laparoscopic cholecystectomy procedures, postoperative pain intensity might be high due to intraabdominal pressure, irritation from gases, and the insertion of trocars [1]. Reducing postoperative pain enhances the ability to breathe deeply and cough effectively, thereby protecting pulmonary functions. This leads to better oxygenation and probable preservation of positive myocardial oxygen balance, which in turn reduces hospital stay and cost and increases patient satisfaction [2–4].

Dexmedetomidine, a highly selective *α*_2_ adrenoreceptor agonist, has anxiolytic, sedative, anesthetic, and analgesic properties. It has limited side effects in terms of respiratory depression [5]. Because of these favorable properties, it is commonly used in a wide variety of procedures [6–11].

Ketamine hydrochloride, a well-known anesthetic agent, has been in clinical use for more than four decades. Its antinociceptive-hypnotic effects are most likely the result of the noncompetitive antagonism at the N-methyl-D-aspartate (NMDA) receptor of the central nervous system [12–14]. Ketamine is used as an analgesic in low doses and as an anesthetic in high doses [14]. It is suggested that ketamine maintains analgesia and reduces postoperative opioid use, whether used alone or in combination with other anesthetic agents [13–18].

Ketamine and another rarely used *α*_2_ adrenoceptor agonist, clonidine, have been compared for their effects on reducing pain and anesthesia requirements. According to our literature research studies, no similar studies have been found that compare ketamine and dexmedetomidine for the same effects [19, 20].

In our study, we aimed to compare the postoperative analgesic and adverse effects of dexmedetomidine and ketamine combined with total intravenous anesthesia (TIVA) in patients undergoing elective laparoscopic cholecystectomy procedures.

## 2. Materials and Methods

After approval from the ethics committee (number: 2007–8/3, date: 1st May 2007) and written informed consent from 90 patients between 18 and 50 years of age, they with American Society of Anesthesiologists (ASA) physical status I-II and undergoing elective laparoscopic cholecystectomy procedures were included in this prospective randomized controlled study. Patients with ASA physical status ≥3, emergency operations, allergy to the drugs used in the study, drug and/or alcohol abuse, refusal to join the study, mental vs psychiatric disorders, and out of age ranges were not included in the study. Patients whose operations turned into open cholecystectomy were excluded from the study. Upon arrival to the operating room, routine monitorization for heart rate (HR), noninvasive arterial blood pressure (systolic arterial pressure (SAP), diastolic arterial pressure (DAP), mean arterial pressure (MAP)), and peripheral oxygen saturation (SpO_2_) were made, and all hemodynamic parameters were recorded preoperatively, during induction and endotracheal intubation, at the beginning of the operation, intraoperatively every 15 minutes, during extubation, and in the recovery unit. SAP <80 mmHg or MAP <60 mmHg (MAP <25% baseline level) was considered as hypotension and MAP >25% of the baseline level was considered hypertension; HR >25% of the baseline level was considered tachycardia and HR < 45 beat/min was considered as bradycardia. No premedication was administered. After preoxygenation, general anesthesia was induced with remifentanil (1 *μ*g/kg), propofol (2 mg/kg), and rocuronium (0.6 mg/kg). After endotracheal intubation, patients were ventilated with a mixture of 33% oxygen and 67% air to achieve end-tidal carbon dioxide (ETCO_2_) at 35–40 mmHg. A standard disposable sensor was applied to the patient's forehead for bispectral index (BIS) monitorization. TIVA was started with an intravenous (IV) infusion of remifentanil (0.1–0.5 *μ*g/kg/min), propofol (8–12 mg/kg/hour), and rocuronium (5–10 *μ*g/kg/min). Propofol infusion rates were adjusted to maintain a BIS value between 45 and 55. Rocuronium was administered after receiving a 1-2 twitch response to a train of four (TOF). The remifentanil infusion rate was increased when hypertension and tachycardia were observed. Patients were randomized into three groups. Group KETA received ketamine infusion of 10 *μ*g/kg/min after a 0.5 mg/kg IV bolus dose of ketamine, and group DEX received dexmedetomidine infusion at 0.5 *μ*g/kg/hour, which was administered after a 1 *μ*g/kg IV bolus dose of dexmedetomidine 10 minutes before skin incision. An infusion of normal saline was added to TIVA in the control group (group C). All infusions were stopped at the end of the surgery. The neuromuscular blockade was reversed with 0.07 mg/kg neostigmine +15 *μ*g/kg atropine. All patients were transferred to the postanesthesia care unit (PACU).

Recovery was evaluated using the quality of recovery (QoR) score [15] (Table 1). Postoperative pain was assessed using the visual analogue scale (VAS) (VAS:0–10, where 0 = no pain, and 10 = worst possible pain). Before surgery, all patients were given a short education about VAS. VAS pain scores were evaluated in the recovery unit and postoperatively at the 4^th^, 8^th^, 24^th^, and 48^th^ hours. Postoperative analgesia was provided with IV patient-controlled analgesia (PCA) pumps (1 mg bolus morphine, 5 min lockout time). If VAS pain scores were evaluated higher than 4, dexketoprofen 50 mg IV was used as rescue analgesia. Patients were observed for side effects (nausea, vomiting, sedation, and bradypnea) in the PACU and discharged to their clinics when their Aldrete score [21] was greater than 8. All patients were followed-up for 48 hours in their clinics.

### 2.1. Statistical Analysis

The statistical analysis was performed using a standard SPSS software package. No sample size calculation methods were used. One-way analysis of variance (ANOVA), Bonferroni, Kruskal–Wallis, Mann–Whitney *U,* and chi-square tests were employed as appropriate, and *P* values ≤ 0.05 were considered statistically significant.

## 3. Results

Demographic characteristics, duration of anesthesia, and surgery were similar among the three groups (Table 2).

Hemodynamic parameters were also found similar between groups.

Propofol consumption was found to be lower in group KETA than in group DEX and group C (*P*=0.034, *P* < 0.001) (Table 2). The requirement for intraoperative of remifentanil was significantly lower in group KETA than in group DEX and group C (*P*=0.049, *P* < 0.001) (Table 2).

The recovery quality score and time of the first oral intake were found to be similar between groups.

Postoperative first analgesic time was found to be longer in group KETA than in group DEX and group C (*P* < 0.001, *P* < 0.001), and it was found to be longer in group DEX than in group C (*P* < 0.001) (Table 3).

The amount of morphine consumption in the postoperative recovery unit and at the 4^th^, 24^th^, and 48^th^ hours was found to be greater in group C than in group KETA and greater in group DEX than in group KETA (*P* < 0.001, *P* < 0.001, *P* < 0.001 for all followed-up times) (Table 3).

Rescue analgesic drug requirement was significantly higher in group C than that of group KETA and group DEX (*P* < 0.001, *P* < 0.001) (Table 3).

No statistically significant difference was found with respect to side effects between groups.

## 4. Discussion

This prospective randomized controlled study demonstrated that the administration of preemptive ketamine reduced the requirement for anesthetic and analgesic agents. Moreover, it was detected that both ketamine and dexmedetomidine prolonged the first analgesic requirement time and reduced postoperative VAS pain scores and morphine consumption.

The sedative effects of dexmedetomidine, a potent *α*_2_-adrenergic agonist, are in part mediated through an increase in parasympathetic outflow and a decrease in sympathetic outflow from the locus ceruleus in the brain stem. Its sympatholytic effects are mediated through the activation of negative feedback receptors in the medullary vasomotor center, which results in reduced catecholamine release [5, 8]. Due to the reduction of norepinephrine release and the possible baroreflex activation, bradycardia and hypotension can be observed during dexmedetomidine use. It has been reported that these side effects are related directly to the dose and/or technique of the administration of the agent and that they are caused by a high dose or short loading time [9, 11, 22–24]. At low doses, these side effects might be minimal or lacking [8, 25]. Moreover, without a loading dose or a loading dose given over 20 minutes is suggested to maintain hemodynamic stability [25, 26]. Park et al. [23] also observed a reduction in both heart rate (HR) and blood pressure after an infusion, following a 10-minute loading dose. These authors suggested that this 10-minute loading dose was not enough to be considered a high dose and that these side effects were due to the drug's central sedative effects and a reduction of both sympathetic outflow and catecholamines. However, in Gurbet et al.'s study [25], none of the patients who received intraoperative dexmedetomidine infusion at a rate of 0.5 *μ*g/kg/h developed clinically significant bradycardia, either during surgery or postoperatively. In our study, we used the same dose and also did not observe any hemodynamic instability in our patients.

Ketamine, a noncompetitive NMDA receptor antagonist, is a primary component of TIVA regimens. At low doses, it has numerous favorable effects such as the maintenance of airway reflexes and respiratory drive and a stable HR, blood pressure, and cardiac output [12, 14]. However, it has cardiovascular stimulating and psychomimetic effects at high doses [27]. Bajwa et al. [28] demonstrated that a bolus IV injection of 1 mg/kg ketamine followed by an IV injection of 2.0 mg/kg/h ketamine and 2.0 mg/kg/h propofol maintained stable hemodynamics when compared to an IV infusion of 2.0 mg/kg/h propofol combined with 20 *μ*g/kg/h fentanyl. In our study, an IV 0.5 mg/kg bolus dose and 10 *μ*g/kg/min infusion dose of ketamine was administered and no hemodynamic instability was observed.

Systemic administration of dexmedetomidine has been reported to cause sedative effects and reduce the requirement for anesthetic agents during the perioperative period [11, 24, 26, 29, 30]. Ngwenyama et al. [26] showed that the addition of dexmedetomidine to a propofol-remifentanil infusion during spinal fusion surgery reduced propofol infusion requirements by approximately 30%. Bajwa et al. [29] also used dexmedetomidine as part of a TIVA regimen during scoliosis surgery and found that the amount of propofol required for the desired depth of anesthesia was reduced. Additionally, when compared to a control group, it was reported that in patients monitored with BIS, an IV bolus dose at 1 *μ*g/kg of dexmedetomidine injected over 10 minutes followed by an IV infusion at 0.5 *μ*g/kg/min of dexmedetomidine resulted in a significant reduction in both the desired induction dose of propofol and perioperative mean end-tidal sevoflurane concentrations [23]. In our study, the propofol requirement was also reduced in the dexmedetomidine group.

Ketamine, an anesthetic agent, when used in low doses, reduces the intraoperative requirement for other anesthetics [12, 27]. After an IV ketamine bolus of 0.5 mg/kg was administered for the induction of anesthesia in diagnostic gynecologic laparoscopic surgery, the dose of propofol was found to be reduced [27]. In our study, the required dose of propofol was found to be lower in the ketamine group than in the control group.

Besides its sedative effects, dexmedetomidine also has analgesic effects when included in TIVA regiments; it induces analgesia and decreases opioid requirements during the perioperative periods [8, 11, 22, 25, 31]. An evaluation of dexmedetomidine's analgesic effect in healthy volunteers was conducted by systemic administration of different doses (0.25, 0.50, and 1 *μ*g/kg). It was found that dexmedetomidine had a moderate analgesic effect that was maximized at a 0.5 *μ*g/kg dose [31]. The authors found that the analgesic effect of dexmedetomidine was not dose-dependent; they observed an apparent ceiling effect at the dose of 0.5 *μ*g/kg, which was used in our study. Abdelmageed et al. [8] found that the intraoperative use of dexmedetomidine reduced postoperative cumulative morphine consumption in the first 24 hours. The authors suggested that this result strongly supports the presence of a dexmedetomidine-induced opioid-sparing effect. Similarly, in our study, the use of remifentanil was significantly reduced in the dexmedetomidine group compared to the control group.

It is suggested that postoperative use of dexmedetomidine, as well as intraoperative use, reduces VAS scores and analgesic consumption [8, 9, 11, 24, 25, 32]. Abdelmageed et al. [8] reported lower VAS scores in the first two hours after extubation, longer time of first analgesic requirement and lower cumulative morphine consumption at the 12^th^ and 24^th^ postoperative hours, and lower rescue analgesic requirement in the dexmedetomidine group compared to the control group. Patel et al. [9] reported that the use of dexmedetomidine provided lower pain and agitation scores and a long time of first analgesic requirement, suggesting that dexmedetomidine had a significant analgesic effect. Using an IV 0.2–0.8 *μ*g/kg/h dexmedetomidine infusion during laparoscopic bariatric surgery was found to reduce fentanyl consumption in the postoperative period [24]. Tufanogullari et al. [25] found that dexmedetomidine infusion during total abdominal hysterectomy surgery reduced postoperative morphine consumption. They also suggested that dexmedetomidine administered intraoperatively had specific analgesic properties and provided effective visceral pain relief. Arain et al. [23] also detected that dexmedetomidine use during laparoscopic cholecystectomy reduced VAS scores during the first hour and analgesic consumption in 24 hours in the control group. In our study, we found that dexmedetomidine infusion during the intraoperative period caused a reduction in postoperative VAS scores, PCA morphine consumption, rescue analgesic requirement, and a long time of first analgesic requirement.

Preoperative administration of ketamine should prevent central sensitization and may improve postoperative pain relief [12, 13, 15, 18]. A small dose of ketamine, given before skin incision, was shown to decrease postoperative pain, reduce morphine consumption, and delay analgesia requirement after laparoscopic gynecologic surgery [15]. However, as postoperative analgesia was not improved in patients who received ketamine after skin closure, it was suggested that the timing of ketamine treatment was critical in its analgesic efficacy. Taghinia et al. [33] compared the analgesic effect of a presurgical loading dose (0.5 mg/kg), followed by a continuous infusion (10 *μ*g/kg/min) of ketamine with a single postsurgical dose (0.5 mg/kg). They found a significant reduction in PCA morphine consumption within 48 hours after surgery in the preemptive group. Adam et al. [16] found that in total knee arthroplasty procedures, a preemptive dose of ketamine 0.5 mg/kg IV followed by a 3 *μ*g/kg/min infusion intraoperatively and a 1.5 *μ*g/kg/min infusion for 48 hours postoperatively reduced PCA morphine consumption at a ratio of 35%. Ngwenyama et al. [27] also found that a lower postoperative pain score and a longer time to the first analgesic requirement were observed in the group that was administered 0.5 mg/kg IV ketamine during anesthesia induction. They suggested that this result was due to the preemptive analgesic effect of ketamine. Our study also demonstrated that ketamine reduced postoperative PCA morphine consumption.

Because of the sedative effects of *α*_2_ agonists, dexmedetomidine may prolong anesthetic recovery time when used together with other anesthetics [5]. Arain et al. [23] found that BIS levels were significantly reduced 10 minutes after dexmedetomidine infusion. In a study that compared dexmedetomidine and remifentanil use during video laparoscopic surgery, the time to postoperative extubation and orientation was longer in the dexmedetomidine group, but there was no significant difference in the length of stay in the postoperative unit between the two groups [7]. It was reported that dexmedetomidine did not affect extubation time but modified Aldrete scores were increased [9, 11]. Patel et al. [9] found significant sedation during the first two postoperative hours in the cases where dexmedetomidine was used. In this study, modified Aldrete scores were found to be higher in the dexmedetomidine group compared to the control group 30 minutes after extubation; however, after two hours, no significant difference was observed between these groups. The authors reported that because dexmedetomidine affected recovery time, close monitorization should have been conducted during the first few hours postoperatively. Although some authors observed sedation after dexmedetomidine use, they did not observe any respiratory failure presenting as desaturation or tachypnea [11, 23, 32]. Tufanogullari et al. [25], however, did not observe clinically significant sedation in any patients receiving an intraoperative IV dexmedetomidine infusion at a rate of 0.5 *μ*g/kg/h. Furthermore, we did not observe significant sedation during the postoperative period with this same infusion dose of dexmedetomidine. Because we did not observe any desaturation or respiratory failure, the recovery quality of patients was considered normal.

It was reported that intraoperative use of dexmedetomidine reduced side effects like nausea, vomiting, and pruritus [8, 24]. Adam et al. [16] used an intraoperatively dexmedetomidine infusion of three different doses (0.2, 0.4, and 0.8 *μ*g/kg/h) and detected that postoperative use of antiemetics was reduced. These authors suggested that the use of desflurane in addition to dexmedetomidine caused a lower incidence of nausea. Abdelmageed et al. [8] found that intraoperative use of dexmedetomidine reduced pruritus; they suggested that this was due to the analgesic effects of dexmedetomidine, which reduced the need for opioid consumption. In our study, we did not observe any significant side effects such as nausea, vomiting, or pruritus with the use of dexmedetomidine.

High dose of ketamine can cause sedation and drowsiness [12, 14], but these effects and negative effects on QoR are not observed in low doses [15]. In our study sedation, sleepiness and negative effects on QoR were not observed with the low-dose ketamine. Besides sedation and drowsiness, further neuropsychiatric effects such as hallucinations, psychiatric disturbances, unpleasant dreams, diplopia, blurred vision, nystagmus, or dysphoria might be seen during ketamine use [12, 14]. These side effects limit the clinical usefulness of ketamine [12]. High doses of ketamine (>2 mg/kg IV) and rapid IV administration (>40 mg/min IV) are associated with psychotomimetic effects [12, 34]. However, side effects are rare with a reduced dose of IV ketamine ranging from 0.15 to 0.5 mg/kg [12]. Adam et al. [16] reported that in the cases where a low dose of ketamine infusion was used during the preoperative and postoperative periods, no significant difference was found in side effects compared to the control group. Ngwenyama et al. [27] also found no significant difference with respect to nausea and hallucinations between the control group and the group in which 0.5 mg/kg of ketamine was administered. In the present study, ketamine was used in low doses and did not cause significant neuropsychiatric effects.

In a study by Nitta et al. [19], where oral clonidine (4 *μ*g/kg) was combined with ketamine (10 mg IV for anesthesia induction followed by an IV infusion at 2 mg/kg/h), postoperative PCA morphine consumption was found to be significantly reduced. Khafagy et al. [20] also found that coadministration of clonidine as an adjuvant to TIVA significantly reduced intraoperative propofol and fentanyl consumption when compared with ketamine.

After a review of 89 papers by Barends et al., they found that dexmedetomidine was a promising alternative to midazolam for use in procedural sedation [35]. In our study, we did not compare these two drugs, but we found ketamine superior to dexmedetomidine.

Görges et al. found some transient reduction of QT interval with rapid bolus administration of dexmedetomidine combined with standardized propofol and remifentanil TIVA in pediatric patients in a retrospective study, but we did not see any cardiac side effects with dexmedetomidine with ASA I-II physical status in adult patients [36].

Hwang et al. found dexmedetomidine superior to remifentanil for postoperative pain management when combined with propofol TIVA in spinal surgery [37]. In our study, dexmedetomidine was used with remifentanil and propofol TIVA.

Koruk et al. compared the effect of dexmedetomidine and ketamine combined with propofol TIVA during transcatheter atrial septal defect closure procedures in 9 pediatric patients, and they found that both of them were well tolerated, but the recovery time was significantly shorter with dexmedetomidine [38]. In our study, the recovery quality score was found to be similar.

Although there are some limitations like not being a double-blinded study, with a low number of patients, with different surgeons and different anesthesiologists, this study is very original due to being the first one comparing the effects of dexmedetomidine and ketamine combined with a TIVA regimen in adult patients.

For better and more reliable results, future studies with double-blinded, with more number of patients, with pediatric or geriatric patients, with a special group of patients, with regional anesthesia, sedation, with different kinds of agents and procedures, with the same surgeon are needed to be done.

As a result of this original study, we strongly believe that combining dexmedetomidine or ketamine infusion with a TIVA regimen during the intraoperative period of laparoscopic cholecystectomy procedures is useful for reducing postoperative pain and analgesic requirements without significant adverse effects. It should be mentioned that ketamine seems to be a more effective analgesic than dexmedetomidine.

## Figures and Tables

**Table 1:** The quality of recovery (QoR) score [15].

	Not at all	Some of the time	Most of the time
Having a feeling of general well-being	0	1	2
Having a feeling of support from others	0	1	2
Able to understand instructions and advice. Not being confused	0	1	2
Able to look after personal toilet and hygiene unaided	0	1	2
Able to pass urine and have trouble with bowel function	0	1	2
Able to breathe easily	0	1	2
Free from headaches, backache, or muscle pains	0	1	2
Free from nausea, dry-retching, or vomiting	0	1	2
Free from experiencing severe pain or constant moderate pain	0	1	2

**Table 2:** Demographic characteristics, durations of anesthesia and surgery, and total amounts of propofol and remifentanil used during total intravenous anesthesia (mean ± SD/number).

	Group KETA (*n* = 30)	Group DEX (*n* = 30)	Group C (*n* = 30)	*P*
Age (year)	37.90 ± 7.69	36.40 ± 7.82	36.37 ± 8.14	NS
Gender (M/F)	11/19	15/15	12/18	NS
ASA (I/II)	21/9	19/11	20/10	NS
Weight (kg)	74.00 ± 13.24	72.70 ± 10.66	75.73 ± 12.14	NS
Height (centimeter)	170.53 ± 10.52	170.23 ± 10.68	168.04 ± 8.01	NS
Duration of anesthesia (min)	102.93 ± 28.33	94.73 ± 19.25	100.56 ± 21.28	NS
Duration of surgery (min)	86.87 ± 20.55	80.46 ± 20.14	87.50 ± 21.02	NS
The total amount of propofol administered (mg)	782.08 ± 168.12^+#^	858.85 ± 150.59^§^	934.10 ± 155.74	^+^ *P*=0.034
				^#^ *P* < 0.001
				^§^ *P*=0.048
The total amount of remifentanil administered (*μ*g)	672.55 ± 159.78^+#^	754.91 ± 169.12^§^	830.44 ± 163.14	^+^ *P*=0.001
				^#^ *P*=0.049
				^§^ *P*=0.042

KETA, ketamine group; DEX, dexmedetomidine group; C, control group; M, male; F, female; ASA, American Society of Anesthesiology; NS, not significant. ^+^Compared with group DEX, ^#^compared with group C, ^§^compared with group C.

**Table 3:** Postoperative data (mean ± SD).

	Group KETA (*n* = 30)	Group DEX (*n* = 30)	Group C (*n* = 30)	*P*
The first analgesic requirement time (min)	127.47 ± 32.52^+#^	59.13 ± 3.18^§^	26.33 ± 9.10	^+^ *P* < 0.001
				^#^ *P* < 0.001
				^§^ *P* < 0.001

VAS				
Recovery unit	3.17 ± 0.46^+#^	4.60 ± 0.68^§^	6.33 ± 1.47	^+^ *P* < 0.001
				^#^ *P* < 0.001
				^§^ *P* < 0.001
4 h after operation	3.37 ± 0.77^#^	4.47 ± 0.51^§^	4.97 ± 0.85	^#^ *P* < 0.001
				^§^ *P*=0.001
8 h after operation	3.77 ± 0.97^+#^	4.43 ± 0.77^§^	4.80 ± 0.76	^+^ *P*=0.009
				^#^ *P* < 0.001
				^§^ *P* < 0.001
24 h after operation	3.90 ± 0.66^+#^	3.47 ± 0.51^§^	3.90 ± 0.80	^+^ *P* < 0.001
				^#^ *P* < 0.001
				^§^ *P*=0.005
48 h after operation	3.0 ± 0.53^#^	3.07 ± 0.25^§^	2.93 ± 0.37	^+^ *P* < 0.001
				^#^ *P*=0.009
				^§^ *P* < 0.001

The total amount of morphine consumption				
Recovery unit	6.33 ± 1.71^+#^	10.00 ± 1.50^§^	17.60 ± 6.41	^+^ *P* < 0.001
				^#^ *P* < 0.001
				^§^ *P* < 0.001
4 h after operation	3.30 ± 1.26^+#^	7.40 ± 1.67	12.27 ± 3.54	^+^ *P* < 0.001
				^#^ *P* < 0.001
8 h after operation	1.27 ± 0.52	2.23 ± 0.68^§^	2.73 ± 1.59	^§^ *P*=0.002
24 h after operation	1.10 ± 0.48^+#^	0.93 ± 0.37^§^	1.10 ± 0.31	^+^ *P* < 0.001
				^#^ *P* < 0.001

48 h after operation	0.17 ± 0.38^+#^	0.83 ± 0.46^§^	0.97 ± 0.41	^+^ *P* < 0.001
				^#^ *P* < 0.001
				^§^ *P*=0.005
Rescue analgesic drug (*n*) (%)	9 (30%)^#^	15 (50%)^§^	28 (93.3%)	^#^ *P* < 0.001
				^§^ *P* < 0.001

KETA, ketamine group; DEX, dexmedetomidine group; C, control group; NS, not significant. ^+^Compared with group DEX, ^#^compared with group C, ^§^compared with group C.

## Data Availability

Access to data is restricted.
